# Identifying keys to success in reducing readmissions using the ideal transitions in care framework

**DOI:** 10.1186/1472-6963-14-423

**Published:** 2014-09-23

**Authors:** Robert E Burke, Ruixin Guo, Allan V Prochazka, Gregory J Misky

**Affiliations:** Department of Veterans Affairs Medical Center, Eastern Colorado Health Care System, 1055 Clermont St, Denver, CO 80220 USA; Division of General Internal Medicine, Department of Medicine, University of Colorado School of Medicine, Denver, USA; Department of Biostatistics and Informatics, University of Colorado School of Public Health, Denver, USA; Hospital Medicine Section, Division of General Internal Medicine, University of Colorado School of Medicine, Denver, USA

**Keywords:** Readmissions, Framework, Interventions

## Abstract

**Background:**

Systematic attempts to identify best practices for reducing hospital readmissions have been limited without a comprehensive framework for categorizing prior interventions. Our research aim was to categorize prior interventions to reduce hospital readmissions using the ten domains of the Ideal Transition of Care (ITC) framework, to evaluate which domains have been targeted in prior interventions and then examine the effect intervening on these domains had on reducing readmissions.

**Methods:**

Review of literature and secondary analysis of outcomes based on categorization of English-language reports published between January 1975 and October 2013 into the ITC framework.

**Results:**

66 articles were included. Prior interventions addressed an average of 3.5 of 10 domains; 41% demonstrated statistically significant reductions in readmissions. The most common domains addressed focused on monitoring patients after discharge, patient education, and care coordination. Domains targeting improved communication with outpatient providers, provision of advanced care planning, and ensuring medication safety were rarely included. Increasing the number of domains included in a given intervention significantly increased success in reducing readmissions, even when adjusting for quality, duration, and size (OR per domain, 1.5, 95% CI 1.1 - 2.0). The individual domains most associated with reducing readmissions were Monitoring and Managing Symptoms after Discharge (OR 8.5, 1.8 - 41.1), Enlisting Help of Social and Community Supports (OR 4.0, 1.3 - 12.6), and Educating Patients to Promote Self-Management (OR 3.3, 1.1 - 10.0).

**Conclusions:**

Interventions to reduce hospital readmissions are frequently unsuccessful; most target few domains within the ITC framework. The ITC may provide a useful framework to consider when developing readmission interventions.

**Electronic supplementary material:**

The online version of this article (doi:10.1186/1472-6963-14-423) contains supplementary material, which is available to authorized users.

## Background

Unsafe transitions of care from the hospital to the community are common and are frequently associated with post-discharge adverse events, including hospital readmission [1]. While not all hospital readmissions are preventable, the volume of patients readmitted (nearly one in five Medicare patients by 30 days post-discharge) and costs associated with readmissions ($26-44 billion per year spent by Medicare) make remediating unsafe transitions essential [2].

However, best practices to cost-effectively reduce readmissions are not well-elucidated [3]. A previous systematic review of interventions to reduce hospital readmissions did not identify an intervention or bundle of interventions that reliably reduced readmissions, despite well-conducted individual trials that have reduced readmission rates [4]. In that review, the authors constructed a simple temporal taxonomy to categorize interventions into pre-discharge, post-discharge, and “bridging” interventions. We hypothesize that a taxonomy focused on individual activities that lead to safer transitions of care may provide new insights into why some interventions are successful and many others are not.

The Ideal Transition of Care (ITC) framework (Additional file 1: Figure S1) proposes 10 domains to consider in ensuring safe transitions of care, based upon expert guidelines, critical analysis of the literature, and clinical experience [5]. The ITC has been proposed as a method for analyzing failures and guiding new interventions in transitions of care, as well as creating process measures to monitor the quality of care transitions.

We had four related research aims in this study: 1) to establish how frequently each of the ten ITC domains have been utilized in prior interventions; 2) to discover how frequently prior interventions met with success in reducing readmissions; 3) to examine the relationship between each of the ten ITC domains individually with success in reducing readmissions; and 4) to evaluate the relationship between the total number of ITC domains included in an intervention and successful readmission reduction. Thus, we conducted a comprehensive review of the literature to identify prior interventions intended to reduce hospital readmission, and categorized them according to the ten ITC domains for our secondary analysis.

## Methods

### Review of the literature

We conducted a search of MEDLINE, EMBASE, Web of Science, and the Cochrane Library for English-language reports published between January 1975 and October 2013 looking for prospective interventions to reduce readmissions (Additional file 1: Figure S1). The MEDLINE search was carried out in a similar way to a prior systematic review [4], using the following combinations of Medical subject Heading (MeSH) keywords: (“Hospitalization” [Mesh] OR “Patient Discharge [Mesh] OR “Patient Readmission” [Mesh] OR readmission [All Fields] or post discharge [All Fields] OR postdischarge [All Fields] or intervention [All Fields]) AND (“Continuity of Patient Care” [Mesh] OR transition* [All Fields] or coordination [All Fields] OR (“patient readmission” [Mesh] AND “patient discharge” [Mesh]) OR (rehospitali* [title] OR readmi* [title]). We reviewed reference lists of studies we selected for full-text review to identify any additional studies.

Studies were included for full-text review if the abstract indicated the primary objective of the study was to prospectively evaluate the efficacy of a given intervention to reduce readmission rates in an intervention cohort, compared to a nonintervention cohort. We included both interventions for patients with specific disease states and those targeting all discharged patients regardless of disease state. We elected to include studies with endpoints longer than thirty days as many of the domains in the ITC could be delivered over longer time periods and our intent was to evaluate their efficacy overall when included in an intervention, rather than at a single time point. Randomized controlled trials and observational designs were eligible for inclusion.

We excluded retrospective studies, interventions using disease-specific interventions to readmission reduction (such as measurement of brain natriuretic peptide as a method to reduce readmissions in congestive heart failure), or interventions consisting solely of medication titration (such as increasing the dose of an ACE inhibitor in heart failure patients and measuring rehospitalizations as an outcome). Interventions were eligible for inclusion if a disease-specific population was studied but an intervention that was applicable to other disease states was used. We also excluded studies of exclusively pediatric, obstetric, surgical, or psychiatric populations (if the primary focus was on psychiatric readmissions). In cases of multiple reports of the same study or intervention, the earliest publication reporting results of the intervention (if not a pilot study) was used. Two reviewers (Dr. Burke and Dr. Misky) screened all abstracts, and retained relevant articles for full-text review. We included studies for full-text review when the abstract did not clearly indicate whether the inclusion criteria were met.

**Figure 1:**
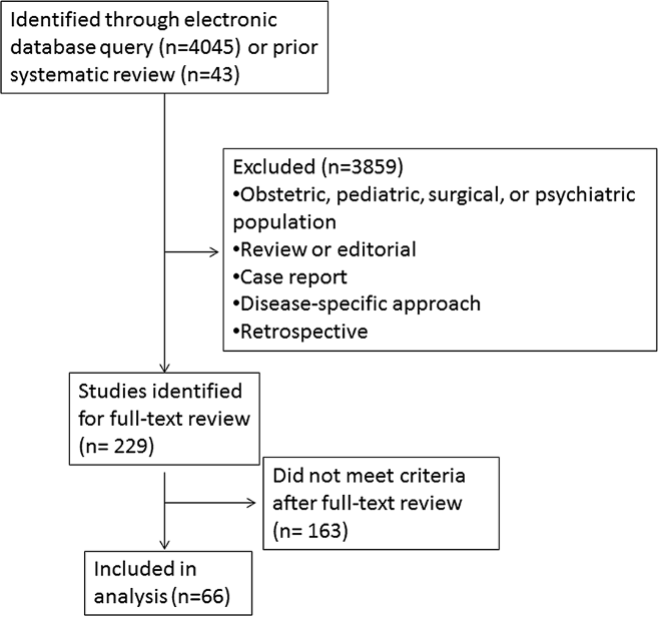
**Selection of studies.** Legend: Selection of studies after application of inclusion and exclusion criteria is shown.

The full text of selected articles was independently reviewed by two reviewers for inclusion and exclusion criteria, and the final list of included articles was reached through discussion and consensus. Studies in which we were unable to identify which domains were targeted were excluded at this stage. Our final cohort of studies included 39 studies from a prior systematic review [4], as well as 27 new studies not included in this review (Figure 1).

### Categorizing into ITC domains

The two reviewers first met to discuss the Ideal Transition of Care framework and review the salient features within each domain. Then, their assessments of the domains included in several papers excluded from the final analysis were compared to identify areas of disagreement and resolve differences. Each intervention included in our final analysis was then independently read by each reviewer in detail to assess and record which of the 10 domains of the Ideal Transition of Care were included in the intervention (graded as present or absent). In case of disagreement between reviewers about whether a domain was included in a particular study, we counted the domain as present if at least one of the reviewers marked it present (Table 1).

**Table 1:** **Details of studies included in the analysis**

Study	Total # domains	Disease specific	Readmissions	Duration (days)	Size
**Randomized Controlled Studies**					
Balaban 2008 [6]	7	All	NS	31	96
Braun 2009 [7]	1	All	NS	30	309
Coleman 2006 [8]	8	All	All-cause	30	750
Dudas 2001 [9]	2	All	NS	30	221
Dunn 1994 [10]	1	All	NS	180	59
Evans 1993 [11]	4	All	All-cause	30	835
Forster 2005 [12]	3	All	NS	30	620
Jaarsma 1999 [13]	3	CHF	NS	30	179
Jack 2009 [14]	8	All	All-cause*	30	738
Koehler 2009 [15]	5	All	All-cause	30	41
Kwok 2004 [16]	4	COPD	NS	28	149
McDonald 2001 [17]	5	CHF	NS	30	70
Naylor 1994 [18]	7	All	All-cause	42	142
Rainville 1999 [19]	3	CHF	Disease-specific	30	34
Wong 2008 [20]	1	All	NS	30	332
Atienza 2004 [21]	5	CHF	All-cause	365	338
Blue 2001 [22]	5	CHF	All-cause	365	165
Bourbeau 2003 [23]	2	COPD	All-cause	365	191
Chaudry 2010 [24]	2	CHF	NS	180	1653
Cline 1998 [25]	4	CHF	NS	365	190
DeBusk 2004 [26]	3	CHF	NS	365	462
Doughty 2002 [27]	4	CHF	All-cause	365	197
Ekman 1998 [28]	4	CHF	NS	180	158
Gillespie 2009 [29]	4	All	NS	365	368
Holland 2005 [30]	4	All	NS	180	872
Kasper 2002 [31]	5	CHF	All-cause	365	200
Kimmelstiel 2004 [32]	5	CHF	Disease-specific	90	200
Koelling 2005 [33]	1	CHF	Disease-specific	180	223
Laramee 2003 [34]	7	CHF	NS	90	287
Ledwidge 2003 [35]	4	CHF	Disease-specific	90	98
Mejhert 2004 [36]	4	CHF	NS	545	208
Murray 2007 [37]	2	CHF	NS	365	314
Nazareth 2001 [38]	5	All	NS	90	362
Peikes 2012 [39]	7	All	All-cause	365	2166
Rich 1995 [40]	6	CHF	All-cause	90	282
Riegel 2002 [41]	5	CHF	Disease-specific	180	358
Stewart 1999 [42]	5	CHF	All-cause	180	200
Stromberg 2003 [43]	4	CHF	All-cause	90	106
Takahashi 2012 [44]	2	All	NS	365	205
Tsuyuki 2004 [45]	3	CHF	NS	180	276
Weinberger 1996 [46]	4	All	NS	180	1396
Marusic [47]	1	All	NS	30	160
**Cohort studies**					
Anderson 2005 [48]	3	CHF	Disease-specific	30	121
Bostrom 1996 [49]	1	All	NS	30	919
Gow 1999 [50]	3	All	NS	28	77
Harrison 2011 [51]	1	All	All-cause	30	30272
Einstadter 1996 [52]	4	All	NS	30	478
Lucas 1998 [53]	1	All	NS	30	285
McPhee 1983 [54]	1	All	NS	30	301
O’Dell 2005 [55]	2	CHF	NS	30	237
Sorknaes 2011 [56]	1	COPD	Disease-specific	28	100
Steeman 2006 [57]	3	All	NS	15	824
Walker 2009 [58]	4	All	NS	30	724
Ohuabunwa [59]	7	All	NS	30	104
**Before-After Comparisons**					
Brown 1997 [60]	5	COPD	All-cause	28	726
Creason 2001 [61]	3	CHF	All-cause	30	293
Dai 2003 [62]	3	CNS	NS	30	283
Dedhia 2009 [63]	4	All	All-cause	30	75
Hess 2010 [64]	2	All	NS	3	362
Houghton 1996 [65]	1	All	NS	28	422
Kramer 2007 [66]	1	All	NS	30	283
Smith 1995 [67]	3	All	All-cause	10	N/A
Mudge 2010 [68]	6	CHF	NS	365	416
Amarasingham [69]	4	All	All-cause	30	1747
Garin [70]	1	CHF	NS	90	363
Graham [71]	1	All	All-cause	30	3295

Legend: Interventions, number of domains included, whether the patient population was disease-specific or not, whether readmissions were statistically significantly reduced (NS = not significant, disease-specific means readmissions were reduced in a specific disease population), duration, and study size are listed. *Composite endpoint of “hospital utilization”.

Intervention size, quality, and duration were recorded by each reviewer. Intervention size was recorded as the size of the total study cohort (including both intervention and control groups) and is reported as a median given distribution of study size. Quality was categorized on a three-point scale, with randomized, prospective trials as the highest-quality category, prospective cohort studies next, and before-after designs as the lowest quality. We found the Cochrane Effective Practice and Organization of Care (EPOC) Group’s Risk of Bias criteria [72] difficult to assess given the limited data provided in previous included publications; this assessment did not contribute significantly to prior analysis of these studies [4]. Duration was recorded as the time point at which the authors reported the study’s primary outcome.

### Analysis of ITC domains

This is a secondary analysis of the publications included above. Success in reducing readmissions was defined as a binary outcome determined by whether there was a statistically significant reduction in readmissions in the intervention group compared to the control group in each of the selected studies. Effect size was not chosen as the outcome for two reasons: first, it was not always reported (for interventions reporting readmissions as a composite outcome, group-specific rates of readmissions were sometimes not reported), and second, we were concerned about the possibility of smaller studies (with large confidence intervals around effect size) unduly influencing our results, where statistically significant reductions in readmissions biases towards larger studies with more power. Bivariate associations between the presence of each of the 10 domains and success in reducing readmissions were examined using Chi-Square tests or Fisher’s exact test if there were small cell counts (<5). The resulting p-values were adjusted for multiple comparisons using a False Discovery Rate (FDR) correction. All comparisons were two-tailed and FDR-adjusted p-values of less than 0.05 were considered to be significant. Unadjusted odds ratios (OR) and their 95% confidence intervals (CI) were also calculated using simple logistic regression.

Simple logistic regression was used to study the crude association between the total number of domains included in an intervention and success in reducing readmissions. We also used multiple logistic regression to study the adjusted association between the total number of domains included and success in reducing readmissions, adjusting for study size, quality, and duration. ORs and their 95% CIs were calculated. All statistical analyses were performed using R 3.0.2 (R Foundation for Statistical Computing, Vienna, Austria). The study was considered exempt by the Colorado Multiple IRB (COMIRB). This study was reviewed and deemed exempt by the Colorado Multiple IRB (COMIRB).

## Results

After application of inclusion and exclusion criteria, 66 articles were included in the final analysis (Additional file 2: Table S1). Median study size was 283 patients (interquartile range, 270). Thirty-five studies (53%) evaluated the primary endpoint at 30 or fewer days following hospital discharge; results of our statistical analyses were similar when comparing studies with primary endpoints of 30 or fewer days with those having endpoints greater than 30 days and thus all studies were analyzed as a single group. Interventions directed at all discharging patients accounted for 52% of included studies, while 41% were studies of heart failure patients exclusively. Overall, 42% of studies demonstrated a statistically significant reduction in readmissions between the intervention and control groups; 61% of these were studies of specific disease processes rather than all discharging patients.

**Figure 2:**
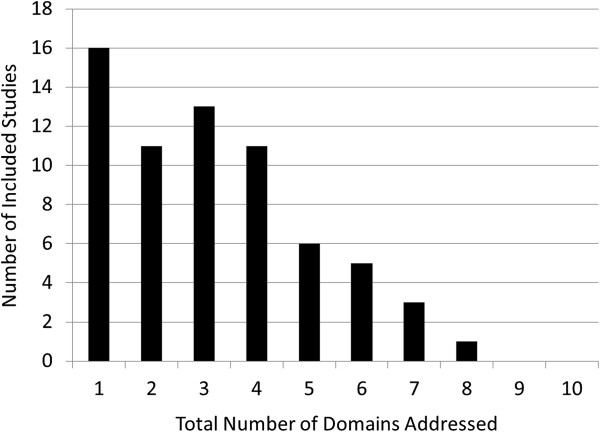
**Number of ITC domains addressed per intervention.** Legend: The distribution of the number of domains of the ITC framework included in each intervention is shown.

**Figure 3:**
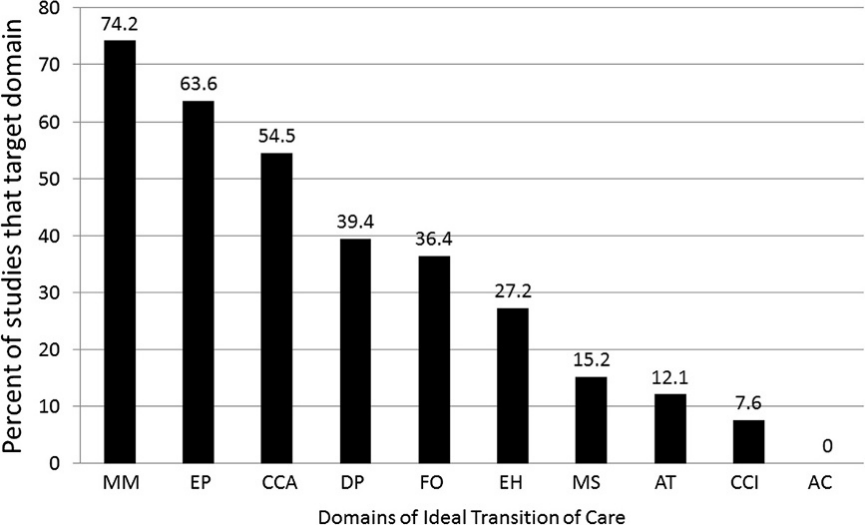
**ITC domains addressed across interventions.** Legend: The percent of interventions that included a particular domain of the ITC framework is shown. **MM** = Monitoring and Managing Symptoms After Discharge; **EP** = Patient Education to Promote Self-Management; **CCA** = Coordinating Care Among Team Members; **DP** = Discharge Planning, **FO** = Outpatient Follow-Up; **EH** = Enlisting Help of Social and Community Supports; **MS** = Medication Safety; **AT** = Accuracy, Timeliness, Clarity, and Organization of Information; **CCI** = Complete Communication of Information; **AP** = Advance Care Planning.

Prior interventions addressed 3.5 domains on average; only 23% addressed five or more (Figure 2). Monitoring and Managing Symptoms after Discharge (included as part of 74% of interventions), Educating Patients to Promote Self-Management (64%), and Coordinating Care among Team Members (55%) were the domains most frequently included as a part of the intervention. Conversely, Advance Care Planning was not included as a part of an intervention in any study, while the two domains concerning information transfer to receiving clinicians and the Medication Safety domain were rarely included (<20%, Figure 3).

In bivariate analysis, the Monitoring and Managing Symptoms after Discharge domain was significantly associated with success in reducing readmissions (OR 8.5 (95% CI 1.8 - 41.1), FDR-corrected p-value = 0.03). Two other domains, Enlisting Help of Social and Community Supports (OR 4.0 (1.3-12.6), FDR-corrected p = 0.07) and Educating Patients to Support Self-Management (OR 3.3 (1.1-10.0), FDR-corrected p = 0.09) showed relatively strong associations with reductions in readmissions (Table 2).

**Table 2:** **The ten domains of the ITC and their association with readmission reduction when part of an intervention**

Domain	Description	p-value*	OR (95% CI)
**Complete Communication of Information (CCI)**	Focuses on the content of the information delivered to the receiving clinician	0.80	2.2 (0.3, 13.9)
**Availability, Timeliness, Clarity, and Organization of Information (AT)**	Highlights if/when this information is received by the receiving clinician, and how it is optimally presented to maximize utility	0.80	1.4 (0.3, 6.2)
**Medication Safety (MS)**	Medication reconciliation across the continuum of care	0.99	1.0 (0.4, 2.7)
**Educating Patients to Promote Self-Management (EP)**	Education to patients and caregivers, using principles of health literacy, teach-back, and encouraging self-advocacy	0.09	3.3 (1.1, 10.0)
**Monitoring and Managing Symptoms after Discharge (MM)**	Multi-modality interventions (telehealth, calls, visits in clinic and/or home), and a responsible clinician to respond to concerns	0.03	8.5 (1.8, 41.1)
**Enlisting Help of Social and Community Supports (EH)**	Adequate assessment of home environment and support and implementing help if needed	0.07	4.0 (1.3, 12.6)
**Advanced Care Planning (AC)**	Establish health care proxy and goals of care	N/A	N/A
**Coordinating Care Among Team Members (CCA)**	Share medical records, communicate with all team members, optimize continuity of providers, formalize handoffs	0.80	1.6 (0.6, 4.2)
**Discharge Planning (DP)**	Emphasizes identifying patient needs prior to discharge, implementing interventions prior to discharge	0.80	1.3 (0.5, 3.5)
**Follow-Up with Outpatient Providers (FO)**	Follow-up with the right provider(s), appropriate time frame, preparation for visit	0.80	1.2 (0.5, 3.4)

*False discovery rate-adjusted p-values are reported.

The number of domains included in an intervention was significantly associated with success in reducing readmissions, even after adjusting for study quality, duration, and size (OR per domain included 1.5, 95% CI 1.1-2.0).

## Discussion

The most important finding of our study for physicians charged with reducing readmissions is that increasing the number of targeted domains within the ITC was associated with significantly increased success in reducing readmissions. In addition, not all domains were associated with equal effect in reducing readmissions. Among the individual domains, systems for Monitoring and Managing Symptoms after Discharge were most associated with successful reduction in readmissions, while Enlisting Help of Social and Community Supports, and Educating Patients to Promote Self-Management may also be efficacious.

Categorizing prior studies in the ITC framework offered important insights into the “state of the science” of readmission reduction. We found most interventions targeting a reduction in hospital readmission published in the literature have not been successful. The 41% overall success rate of published interventions most likely reflects the fact that patients discharged from acute care settings exhibit multiple risk factors for readmission spanning the 10 domains of the Ideal Transition of Care. Since most interventions published targeted a few, similar domains, a correspondingly low success rate of an individual intervention may not be surprising, though our study design limits causal inference. While the ten domains of the ITC framework center on modifiable risk factors for admission, we did not assess how “preventable” readmissions were in included studies.

To the individual clinician, implementing these findings may seem daunting. However, effective multi-domain models exist [8, 14, 18, 39] and nearly all provide options for substantial training. A recurring characteristic of these models is provision of a single health care provider responsive to multiple patient needs, thereby targeting multiple domains of the Ideal Transition of Care. Jack et al. used a “discharge advocate” to provide intensive patient-centered education, discharge planning and post-discharge reinforcement [14]. Likewise, Coleman et al. implemented a “transition coach” to assist patients across health settings and encouraged patients to be active in their own care, while providing them the necessary tools to do so [8]. Similarly, Naylor et al. used an advance practice nurse to manage an individualized patient plan tailored to identified needs, with a focus on patient education and longitudinal collaboration of key providers from hospital admission through two weeks post-discharge [18]. Peikes et al. found success in local care coordination, effectively targeting multiple risk factors for readmission for enrolled patients, and changed their intervention from one that increased readmissions and cost to one that reduced both [39].

However, these models require substantial investment of resources. Clinicians and health care systems with limited resources (particularly those already penalized financially for elevated readmission rates) may struggle to implement these interventions. A key finding from our study is that one option for limiting costs- limiting the number of domains targeted- may not lead to success. A method to risk-stratify patients at the time of discharge, then selectively apply interventions based on this analysis, may maximize efficacy and minimize cost. [3] However, currently available risk prediction models lack accuracy and capture only a global assessment of risk that is difficult to apply to individual patients across highly variable delivery systems. [73] Frameworks similar to the ITC framework may hold promise as tools to better assess individual, *modifiable* risk factors for readmission of recently hospitalized patients, and design interventions to address these risk factors on a case-by-case basis in order to provide tailored, risk-stratified care.

Three domains within the ITC were most associated with success in reducing readmissions. Monitoring and Managing Symptoms after Discharge is plausible as an individual domain most strongly associated with success in reducing readmissions given post-discharge adverse events are common and frequently present with new symptoms. [1] Thus, close clinical monitoring of a recently discharged patient for active symptoms helps ensure effective post-hospital care. Home visits by health care professionals (rather than telemonitoring) appear to be a common theme in several successful interventions [8, 18, 40, 42].

Despite inclusion in fewer than one in four existing interventions, active integration of community and social support networks addressing needs of patients was also associated with success in reducing readmissions. Indeed, this is the intent of Medicare’s $500 million Community-Based Care Transitions Project, part of the Partnership for Patients instituted by the Affordable Care Act. A discharge planning protocol conducted by a social worker to assess living environment and social supports, then engaging community and social service referrals as needed, was the cornerstone of a successful intervention by Evans et al. [11] Several other successful interventions also addressed community supports as a component of a larger intervention [39, 40, 42], indicating the need to address this specific element of a patient’s care transition.

Patient education to promote active involvement in their own care has been a much more commonly targeted domain, though few interventions have assessed the efficacy of this education. Coleman’s transition coach taught patients how to self-manage and to interact with the health care system; benefits were found months after the intervention had concluded [8]. Providing patient education in isolation from other elements and without active patient involvement is likely insufficient to reduce readmissions [13]. Rather, successful interventions focus on engaging the patient to manage their chronic illnesses in an ongoing manner.

These results should be interpreted in the context of the literature reviewed. None of the interventions we reviewed were designed with the ITC framework in mind. As such, our evaluation of whether a domain was present or not represents our best assessment based on our review of these reports and understanding of the ITC. However, implementation of the intervention is infrequently described, and it is possible that the described and actual interventions varied significantly. No study included all ten domains, making our conclusions about the relative influence of inclusion of one domain versus another limited.

Publication bias may play a role in our findings, though we think the strong negative publication record in this regard limits its influence. Published reports may have other biases (academic settings, urban locations) that affect our findings, though analysis of these biases is beyond the scope of this analysis. Our measures of quality were limited to study size, general design, and duration. Other important methodologic constructs such as appropriateness of sampling, data collection, analysis plan, and generalizability were not captured and infrequently reported. While we note inclusion of these elements did not affect findings in prior systematic reviews [4], it is possible their inclusion could have affected our findings.

We excluded pediatric, obstetric, psychiatric, and surgical populations as their reasons for readmission may differ from medical patients. Domains in the ITC may not be independent of one another, but a formal principle components or factor analysis was beyond the scope of our review. We did not use the techniques of a meta-analysis, as the wide variability of the existing literature prevents this level of analysis. We also did not use the reporting standards of a formal systematic review, though we did search systematically for studies that met criteria for analysis. Our approach was necessarily more narrative and thus should be considered hypothesis-generating and requiring further prospective study.

## Conclusions

Improving transitions of care from the hospital to the community requires multifaceted interventions targeting multidimensional risk factors present in patients discharged from the hospital. Until readmission risk factors- individually and collectively- are better understood and assessed, designing interventions to address these multifactorial risk factors using a framework like the ITC may be effective. In addition, incorporating systems actively involving patients in promoting self-management in their care, developing care processes to address active symptom development in the post-discharge period, and providing social and community support for this management merit special inclusion in any intervention. Future work evaluating the role of the Ideal Transition of Care framework in evaluating risk and designing interventions for individual patients may show benefit in providing cost-effective, safe transitional care.

## Electronic supplementary material

Additional file 1: **The Ideal Transition of Care Framework.** Description of data: The Ideal Transition of Care Framework is graphically displayed. (DOCX 112 KB)

Additional file 2: **Categorization of ITC domains by included intervention.** Description of data: Interventions included are listed with each of the ten domains of the ITC judged present (1) or absent (0) by reviewers. (PDF 238 KB)
