# Supplementary material for: Identifying keys to success in reducing readmissions using the ideal transitions in care framework
Source: BMC Health Serv Res. 2014 Sep 23;14:423. doi: 10.1186/1472-6963-14-423 (PMC4180324; doi:10.1186/1472-6963-14-423)
Supplement: Supplementary file 1 — Additional file 1: The Ideal Transition of Care Framework. Description of data: The Ideal Transition of Care Framework is graphically displayed. (DOCX 112 KB) [file 12913_2014_3510_MOESM1_ESM.docx]

eFigure 1 – The Ideal Transition of Care
