# Supplementary material for: Identifying keys to success in reducing readmissions using the ideal transitions in care framework
Source: BMC Health Serv Res. 2014 Sep 23;14:423. doi: 10.1186/1472-6963-14-423 (PMC4180324; doi:10.1186/1472-6963-14-423)
Supplement: Supplementary file 2 — Additional file 2: Categorization of ITC domains by included intervention. Description of data: Interventions included are listed with each of the ten domains of the ITC judged present (1) or absent (0) by reviewers. (PDF 238 KB) [file 12913_2014_3510_MOESM2_ESM.pdf]

Study	Communication	Information	Medications	Education	Symptoms	Supports	Advanced Planning	Coordinating	Discharge Planning	Follow-Up	Total Domains
Amarasingham 2013	0	0	0	1	1	0	0	0	1	1	4
Anderson 2005	0	0	0	1	1	1	0	0	0	0	3
Atienza 2004	0	0	0	1	1	0	0	1	1	1	5
Balaban 2008	1	1	1	0	1	0	0	1	1	1	7
Blue 2001	0	0	0	1	1	1	0	1	0	1	5
Bostrom 1996	0	0	0	0	1	0	0	0	0	0	1
Bourbeau 2003	0	0	0	1	1	0	0	0	0	0	2
Braun 2009	0	0	0	0	1	0	0	0	0	0	1
Brown 1997	0	0	0	1	1	0	0	1	1	1	5
Chaudry 2010	0	0	0	1	1	0	0	0	0	0	2
Cline 1998	0	0	0	1	1	0	0	1	0	1	4
Coleman 2006	1	1	1	1	1	1	0	1	1	0	8
Creason 2001	0	0	0	1	1	0	0	1	0	0	3
Dai 2003	0	0	0	0	0	1	0	1	1	0	3
DeBusk 2004	0	0	0	1	1	0	0	0	0	1	3
Dedhia 2009	0	0	1	1	0	1	0	0	1	0	4
Doughty 2002	0	0	0	1	1	0	0	1	0	1	4
Dudas 2001	0	0	1	0	1	0	0	0	0	0	2
Dunn 1994	0	0	0	0	1	0	0	0	0	0	1
Einstadter 1996	0	0	0	0	1	0	0	1	1	1	4
Ekman 1998	0	0	1	1	1	0	0	1	0	0	4
Evans 1993	0	0	0	0	1	1	0	1	1	0	4
Forster 2005	0	0	0	0	1	0	0	1	1	0	3
Garin 2012	0	0	0	1	0	0	0	0	0	0	1
Gillespie 2009	0	1	1	1	0	0	0	1	0	0	4
Gow 1999	0	0	0	0	0	1	0	1	1	0	3
Graham 2012	0	0	0	0	1	0	0	0	0	0	1
Harrison 2011	0	0	0	0	1	0	0	0	0	0	1
Hess 2010	0	1	0	0	0	0	0	0	1	0	2
Holland 2005	0	0	1	1	0	0	0	1	0	1	4
Houghton 1996	0	0	0	0	0	0	0	0	1	0	1
Jaarsma 1999	0	0	0	1	1	0	0	0	1	0	3
Jack 2009	1	1	1	1	1	1	0	1	1	0	8
Kasper 2002	0	0	0	1	1	1	0	1	1	0	5
Kimmelstiel 2004	0	0	1	1	1	0	0	1	0	1	5
Koehler 2009	0	1	1	1	1	0	0	0	1	0	5
Koelling 2005	0	0	0	1	0	0	0	0	0	0	1
Kramer 2007	0	0	1	0	0	0	0	0	0	0	1
Kwok 2004	0	0	0	1	1	1	0	0	0	1	4
Laramee 2003	0	0	1	1	1	1	0	1	1	1	7
Ledwidge 2003	0	0	0	1	1	0	0	1	0	1	4
Lucas 1998	0	0	1	0	0	0	0	0	0	0	1
Marusic 2013	0	0	1	0	0	0	0	0	0	0	1
McDonald 2001	0	0	0	1	1	0	0	1	1	1	5
McPhee 1983	0	0	0	1	0	0	0	0	0	0	1
Mejhert 2004	0	0	0	1	1	0	0	1	0	1	4

Mudge 2010	1	1	1	1	0	0	0	1	0	1	6
Murray 2007	0	0	1	0	0	0	0	1	0	0	2
Naylor 1994	1	1	0	1	1	1	0	1	1	0	7
Nazareth 2001	0	0	1	1	1	0	0	1	0	1	5
O'Dell 2005	0	0	1	1	0	0	0	0	0	0	2
Ohuabunwa 2013	0	0	1	1	1	1	0	1	1	1	7
Peikes 2012	0	0	1	1	1	1	0	1	1	1	7
Rainville 1999	0	0	1	1	1	0	0	0	0	0	3
Rich 1995	0	0	1	1	1	1	0	1	1	0	6
Riegel 2002	0	0	1	0	1	1	0	1	0	1	5
Smith 1995	0	0	0	0	1	0	0	1	0	1	3
Sorknaes 2011	0	0	0	0	1	0	0	0	0	0	1
Steeman 2006	0	0	0	1	0	1	0	0	1	0	3
Stewart 1999	0	0	1	1	1	1	0	1	0	0	5
Stromberg 2003	0	0	1	1	1	0	0	0	0	1	4
Takahashi 2012	0	0	0	0	1	0	0	1	0	0	2
Tsuyuki 2004	0	0	0	1	1	0	0	0	1	0	3
Walker 2009	0	0	1	1	1	0	0	1	0	0	4
Weinberger 1996	0	0	0	0	1	0	0	1	1	1	4
Wong 2008	0	0	0	0	1	0	0	0	0	0	1
